# Supplementary material for: Molecular genetic characterization of CASEIN KINASE 1-LIKE 12 in Arabidopsis
Source: Plant Signal Behav. 2025 Dec 31;21(1):2610025. doi: 10.1080/15592324.2025.2610025 (PMC12758299; doi:10.1080/15592324.2025.2610025)
Supplement: Supplementary material — Seluzicki_CKL12_SupplementaryFigure1_20251025.docx [file KPSB_A_2610025_SM6823.docx]

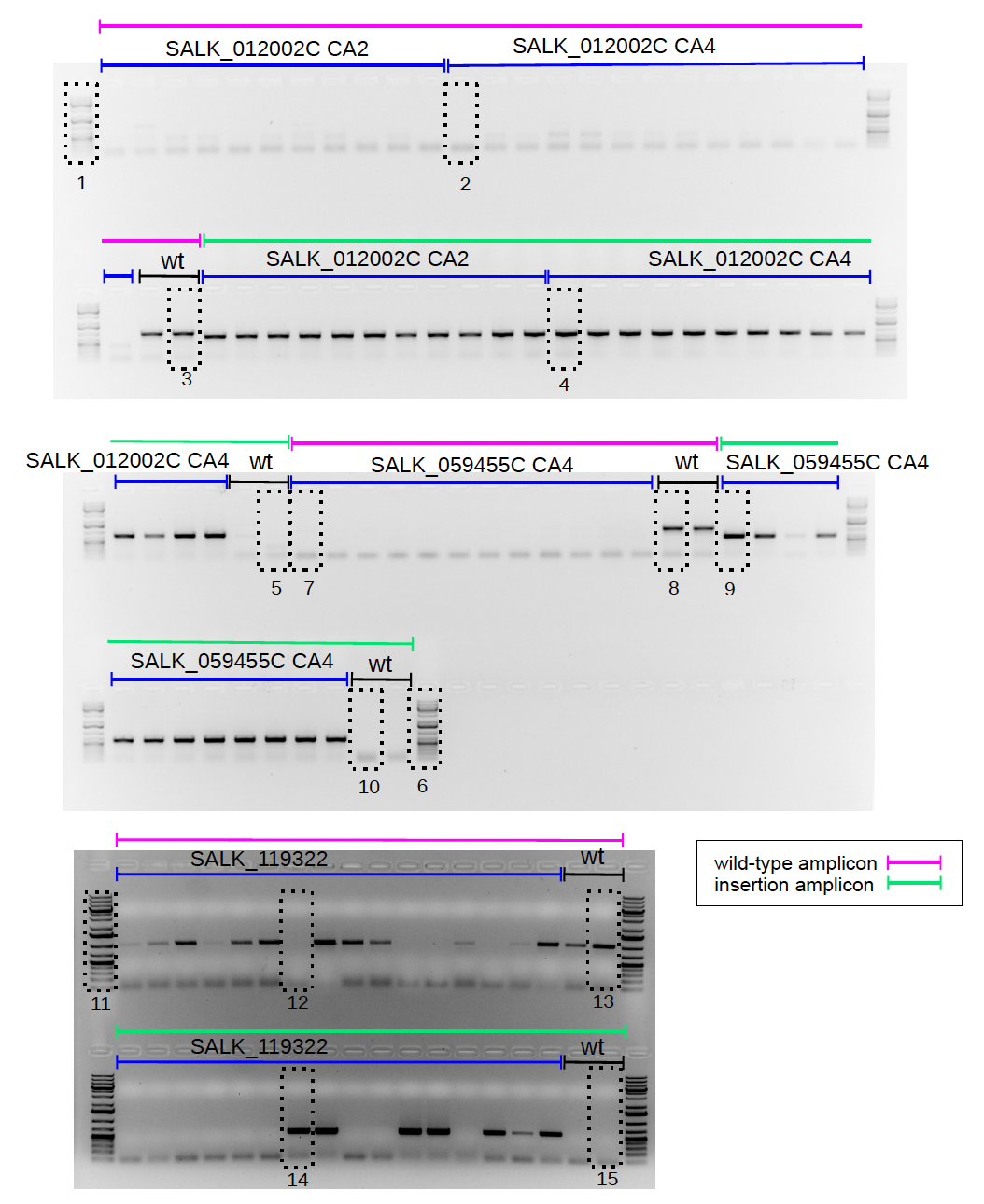


**Supplementary Figure 1: Source data for Figure 2 - Uncropped gel images**

PCR products using genomic DNA from individual plants using primers specific to wild-type or insertion sequences. Sections cropped and reassembled for Figure 2 are boxed. Numbers map to position in Figure 2.
